# Sensory cortex lesion triggers compensatory neuronal plasticity

**DOI:** 10.1186/1471-2202-15-57

**Published:** 2014-05-01

**Authors:** Manfred Depner, Konstantin Tziridis, Andreas Hess, Holger Schulze

**Affiliations:** 1 Experimental Otolaryngology, University of Erlangen-Nürnberg, Waldstrasse 1, 91054 Erlangen, Germany; 2 Institute of Experimental and Clinical Pharmacology and Toxicology, University of Erlangen-Nürnberg, Fahrstrasse 17, 91054 Erlangen, Germany

**Keywords:** Auditory cortex, Shuttle box, Learning, Prepulse inhibition, Deaf hearing, Mongolian gerbil

## Abstract

**Background:**

Lesions to the human brain often cause dramatic impairments in the life of patients because of the very limited capacity of the mammalian nervous system to regenerate. On the other hand, neuronal tissue has a high capacity to reorganize itself so that loss of function due to brain damage may be compensated through neuroplastic reorganization of undamaged tissue in brain regions adjacent or contralateral to the lesion site. In this study we investigated the effect of serial lesions of the auditory cortices (AC) in both hemispheres of Mongolian gerbils on discrimination performance for fast amplitude modulated tones (AM). Healthy animals were trained to discriminate two fast AM, an ability that has previously been shown to critically depend on cortical processing. Their ability to maintain significant discrimination performance was retested after unilateral AC lesion, and again after lesion of the contralateral AC, with 15 days of continuing training in between the two lesions.

**Results:**

After bilateral cortical ablation of both AC and 45 days of training the animals show no change in pure tone detection threshold as measured with modulation of the acoustic startle reflex which has been shown to rely on subcortical structures. In contrast to simultaneous bilateral ablation of AC that results in complete loss of AM discrimination ability in this paradigm we found compensatory plasticity that seems to be triggered by unilateral cortical ablation with subsequent training and that is able to almost fully compensate for the lost cortical functions.

**Conclusions:**

Our results demonstrate that AM discrimination ability that normally depends on AC may be transferred to other brain regions when the brain has time to activate compensatory plasticity between the lesions of the two AC hemispheres. For this process to take place obviously one intact AC hemisphere is needed. This finding may open perspectives for new therapeutic strategies that may alleviate the impairments after multiple ischemic strokes.

## Background

Lesions to the brain, caused by trauma, stroke, or tumors, may lead to a number of functional deficits with dramatic impairment of the life of patients. Type and severity of such impairments strongly depend on the brain region affected by the injury: Whereas lesions of the prefrontal cortex may cause subtle effects that may even remain unnoticed, injuries of the brainstem always lead to severe impairments or even death (cf. [1]).

Although the mammalian nervous system has some capacity to regenerate [2,3], adult neurogenesis in mammals is limited to a few specialized brain regions [4-6]. As a consequence, the regenerative capacity of neuronal tissue falls far short of that of other tissues. This is the reason why most of the impairments resulting from brain lesions still cannot be cured.

On the other hand, neuronal tissue has an extraordinary capacity to reorganize itself. This functional plasticity of the central nervous system allows for some compensation of lost functions through reorganization of undamaged tissue in brain regions adjacent or contralateral to the lesion site (e.g., [3,7-11]). Regardless of the limitations of compensatory neuronal plasticity after brain lesions it has been demonstrated that specific training may foster such compensatory plasticity (e.g., [12]), resulting in improved rehabilitation outcomes in patients with brain damage (e.g., [8]). How effective such compensatory reorganization may be in restoring lost functions depends, first, on the type of function that is lost or impaired and, second, how specialized the cortical region is with respect to this function. For example, there seem to be fundamental differences in the microarchitecture of neuronal connections between the auditory cortices of the two hemispheres [13,14] which can be causative for different sound processing specializations and deficits after unilateral auditory cortex (AC) ablation [15-24]. Therefore, hemispheric specialization in AC may prevent complete compensation of the functional impairment caused by lesion of one hemisphere by the contralateral hemisphere. The same argument applies even more for restrictions of possible compensatory plasticity in subcortical structures for lost cortical functions, especially for functions that seem to rely crucially on intact cortex. In this context we could demonstrate in a previous study [25] that Mongolian gerbils with bilateral lesion of the AC were unable to discriminate between fast amplitude modulations (AM). This was both true for gerbils that received cortical ablation prior to AM discrimination training as well as for animals that were pre-trained to full discrimination performance before AC lesion. On the other hand, animals receiving only unilateral cortical ablations show either impairment or improvement of discrimination performance of fast AM stimuli dependent on the lesion side and size [26]. This difference indicates that the specialization of the two hemispheres might even hinder best performance if they interfere with each other. Nevertheless, each performance gain after a lesion comes with a cost, in this case a loss of frequency modulation discrimination performance [20,21].

Knowing the effects on AM discrimination performance after simultaneous bilateral and unilateral lesions of the AC we now asked the question, what kind of neuronal plasticity can be found after sequential lesioning of both AC as a model of multiple sequential ischemic insults in human patients. We here report the unexpected observation of a compensatory plasticity in other brain regions that seems to be triggered by the first unilateral cortical ablation with subsequent training and that is able to almost fully compensate for the lost cortical functions after removing the second AC.

## Results

### Audiometric controls

**Figure 1:**
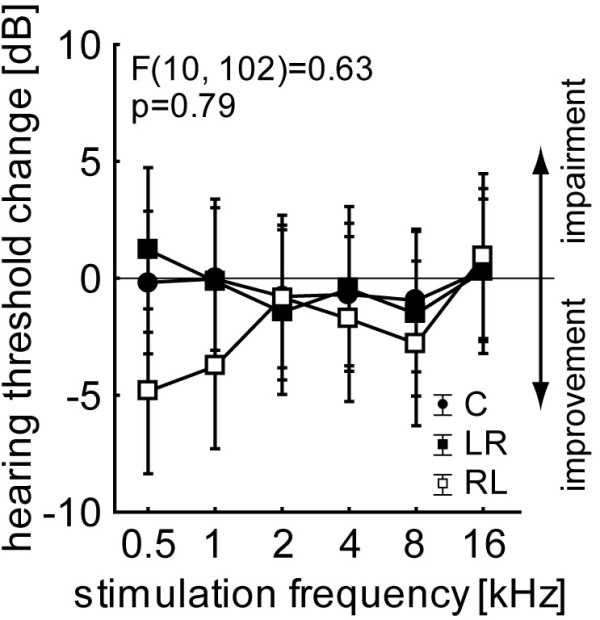
**Mean hearing threshold change of the control group C and the two lesion groups with F interaction statistics.** Whiskers indicate 95% confidence interval.

Animals were tested for a possible general impact of AC lesion on pure tone behavioral audiometry by comparing hearing thresholds prior to the first (i.e., naïve animals without any surgery) and after the last training session (i.e., after 11 weeks of handling and two surgeries; cf. Methods section): Comparing the post vs. pre training hearing threshold differences we found no significant changes, neither within nor across experimental groups (Figure 1; 2-factorial ANOVA: factor group: F(2, 102) = 2.17, p = 0.12; factor frequency: F(5, 102) = 0.64, p = 0.67; interaction: F(10, 102) = 0.63, p = 0.79). In other words, removing both AC had no impact on auditory evoked behavioral responses measured with the brainstem dependent prepulse inhibition (PPI) of the acoustic startle response (ASR; cf. Methods section).

### Quantification and localization of AC lesions

**Figure 2:**
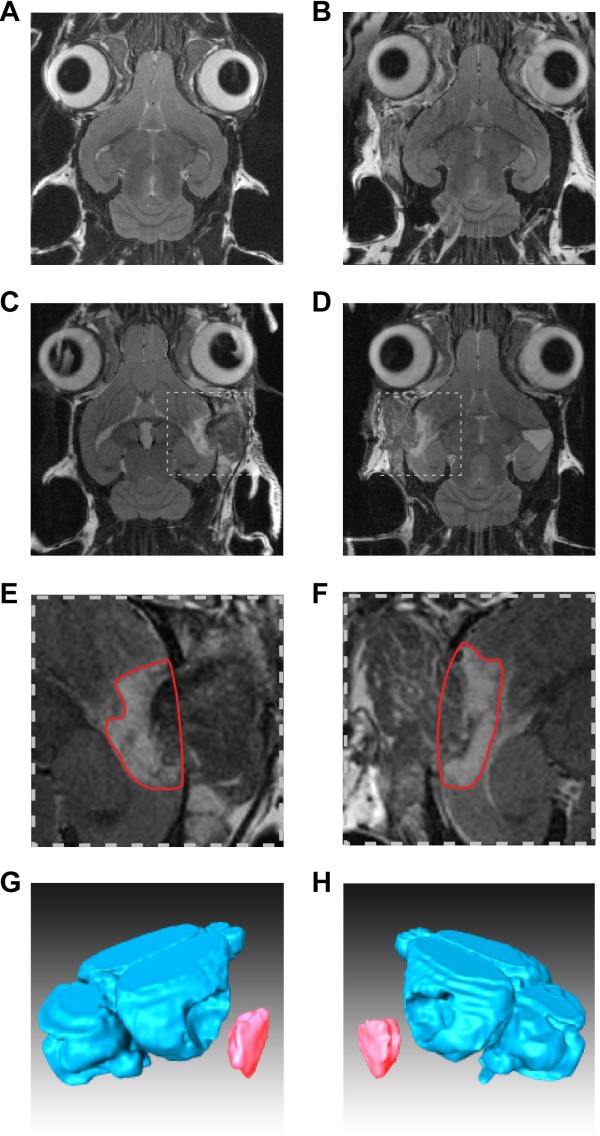
**Structural MRI scans of exemplary animals and lesion reconstruction. A** and **B** Coronal slice of right and left sham lesion in a control animal without any damage to the cortex. **C** and **D** Coronal slice of an animal of the RL group with right (1^st^ lesion) and left (2^nd^ lesion) surgery and thermo-coagulation of the auditory cortices; broken line boxes indicate magnified regions shown below: **E** and **F** Magnified scans from the animal shown above. Red encircled area depicts the lesion boundaries in this specific coronal slice. **G** and **H** Reconstruction of the MRI scanned part of the brain of the lesioned animal shown above. The unhinged volume represents the complete lesion as used for further analysis.

Ablation of AC was performed by thermo-coagulation of parts of the temporal cortex comprising the AC (cf. Methods section). During surgery, localization of AC within the temporal lobe was estimated according to known landmarks of the cortical vasculature. Post lesion MRT scans were used to verify the exact location of the lesion as well as to quantify the ablated volume. An example is given in Figure 2.

Shown are MRT scans of one sham-lesioned control animal after the first and second surgery (Figure 2A and B, respectively), as well as of one animal of the right-left (RL) lesion group (Figure 2C to H). Panels in the left column demonstrate the quantification procedure after lesion of the right AC (1^st^ surgery), panels in the right column show the lesion of the left AC (2^nd^, contralateral surgery). Figure 2C and D show single horizontal slices with the insets comprising the lesioned site shown in higher magnification in Figure 2E and F. The circumscribed areas in these insets delineate the boundaries of the lesion, with an estimate of the former cortical surface by interpolation of the remaining adjacent cortical tissue. These areas across all slices affected by the ablations were used to calculate the lesion volume (cf. Methods section). The rostral border of the hippocampus served as anatomical landmark for the verification of the lesion site: lateral to this landmark lays the 1 kHz iso-frequency contour of the primary auditory cortex (cf. [27]). Animals where lesions did not include the AC or injured subcortical structures (e.g., hippocampus) were discarded from this study (overall 9 out of 23 lesioned animals). Figure 2G and H finally show 3-dimensional reconstructions of the lesioned brain and the removed volume in a representative animal.

The lesion volumes ranged from 4.2 to 21.0 mm^3^ with a grand mean value of 12.2 mm^3^. A statistical comparison of 1^st^ vs. 2^nd^ lesion volumes as well as left vs. right AC lesion volumes revealed no significant differences by a 2-factorial ANOVA (factor 1^st^ lesion side: F(1, 23) = 2.69, p = 0.11; factor lesion number: F(1, 23) = 0.74, p = 0.40; interaction: F(1, 23) = 0.32, p = 0.58). A summary of all lesion volumes is given in Table 1.

**Table 1:** Lesion size in all lesioned animals

**Animal running number**	**Lesion group**	**1**^**st **^**lesion size (mm**^**3**^**)**	**2**^**nd **^**lesion size (mm**^**3**^**)**	**Mean (±STD) of 1**^**st **^**lesion size (mm**^**3**^**)**	**Mean of 2**^**nd **^**lesion size (mm**^**3**^**)**
5	1^st^ lesion left 2^nd^ lesion right(LR)	20.99	12.12	12.42 (±5.58)	14.90 (±2.93)
14		no data	16.6		
16		11.43	12.41		
22		7.15	13.67		
33		16.2	19.96		
35		12.56	10.23		
47		6.17	16.82		
17	1^st^ lesion right 2^nd^ lesion left (RL)	11.08	12.86	10.55 (±4.55)	11.06 (±4.76)
23		6.99	4.22		
30		14.68	16.45		
36		8.85	8.6		
39		18.56	13.63		
46		6.89	7.42		
48		6.82	16.69		

### AM discrimination learning

**Figure 3:**
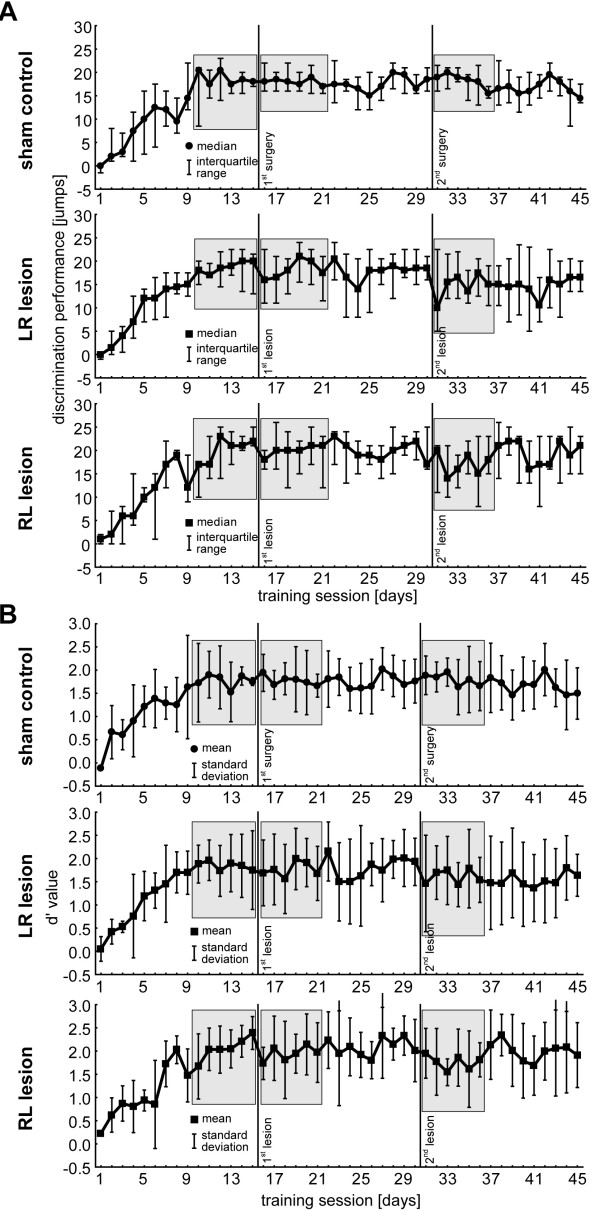
**Discrimination performance in AM discrimination task as a function of training session. A** Top row: Median DP of unified sham control group with interquartile range indicated by the whiskers. Grey boxes indicate the 6-day intervals tested against each other in the main part of the analyses (cf. Figure 4). Center and bottom row: Median DP of the two lesion groups. Note that the animals of both groups are still able to perform the task even after the second lesion, i.e., without auditory cortex. **B** Mean d’ values (±standard deviation) of the three animal groups over the 45 training sessions. Note that for further analysis we chose to use the same 6-day time intervals as in the discrimination performance shown above.

Figure 3 shows a summary of the learning progress in the fast AM discrimination task (CR+: modulation frequency 160 Hz vs. CR-: modulation frequency 320 Hz; carrier frequency 2 kHz each) in all three experimental groups based on two different methods of analysis: Figure 3A shows the median discrimination performance (DP) with the interquartile range as a function of training session in the three animal groups, in the top panel the sham-lesioned control group (C, n = 8) and in the center and bottom panels the data for the lesioned groups left-right (LR, n = 7) and right-left (RL, n = 7), respectively. Figure 3B depicts the respective data of the same animals, but given as the normalized z-score difference values of mean d’ (±standard deviation). While DP is a measure that merely reflects the difference between hit and false alarm responses in total numbers and therefore is potentially prone to individual response bias, the sensitivity index d’ is a measure that is independent of individual response bias. Nevertheless, in this study both measures lead to similar results and identical conclusions.

During the first training phase prior to 1^st^ surgery (training session 1 to 15) all groups showed a continuous improvement in DP leveling off around median values of 18 to 21 jumps at the 10^th^ training sessions (Figure 3A; tested with Friedman ANOVAs for first non-significant plateau: C from day 8 to 15: Χ^2^ (8,7) = 10.73, p = 0.16; LR from day 8 to 15: Χ^2^ (8,7) = 8.05, p = 0.33; RL from day 10 to 15: Χ^2^ (7,5) = 9.16, p = 0.10). There were also no significant differences between all three experimental groups with respect to learning speed (LS; Kruskal-Wallis ANOVA: H(2, 23) = 0.96, p = 0.62) or final DP (i.e., median values across the last 6 training days prior to 1^st^ surgery; Figure 3A 1^st^ grey areas; H(2, N = 138) = 5.18, p = 0.08), so that a bias in the training results based on possible interindividual differences between the training groups prior to the first surgery can be excluded. These results were generally confirmed by the second analysis method investigating the d’ values (Figure 3B). We found the learning leveling off somewhat earlier than in the previous analyses namely between training session 7 and 9 (tested with Tukey post-hoc tests in 1-factorial ANOVAs) but again no difference in the final d’ across the last 6 training sessions (i.e., the same time intervals as above, Figure 3B 1^st^ grey areas) between the three groups could be found (1-factorial ANOVA: F(2, 113) = 2.57, p = 0.08).

**Figure 4:**
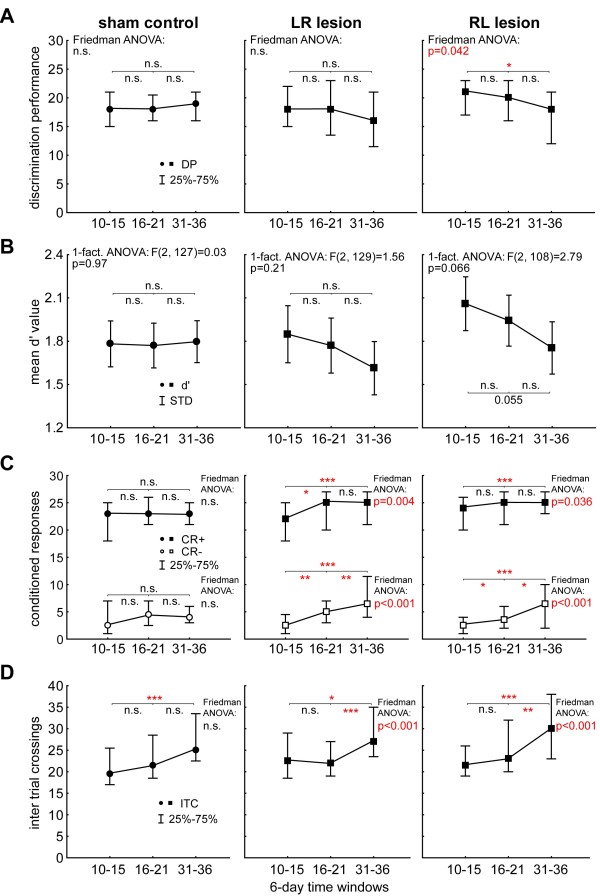
**Statistical intra-group analysis of three different 6-day interval data of all three animal groups. A** Median discrimination performance with interquartile range for C, LR and RL groups with results of the Friedman ANOVAs over all intervals and Bonferroni corrected Wilcoxon tests (tests between two intervals). Only group RL shows a significant loss of DP after the second lesion. **B** Mean d’ values and standard deviations of the three groups over the three 6-day time intervals. These analyses generally confirmed the results of the DP analysis shown above. **C** Median CR+ and CR- responses during the 6-day intervals. Note the significant increase of CR+ and CR- over time in both lesion groups, but not in control group C. **D** Median inter-trial crossing activity. Animals of all groups show an increase of activity in the course of the experiment, but this was more pronounced in the lesioned groups compared to the control group, especially after the second lesion.

After the confirmation that all animal groups started from the same level independent of the analysis method used we compared the different learning parameters of AM discrimination over time. In the control group (Figure 4A, left panel) we found neither any AM discrimination change in 6-day DP by Friedman-ANOVA (comparison of the three 6-day time intervals indicated with grey areas in Figure 3A; ANOVA Χ^2^ (48, 2) = 0.84, p =0.66) nor changes when comparing the last and first training day before and after each surgery by Wilcoxon tests (1^st^ surgery, median day 15 (=pre) (lower, upper quartile) vs. median day 16 (=post) (lower, upper quartile): 18(15, 21) vs. 18(16, 20), p = 0.89; 2^nd^ surgery, median day 30 (=pre) (lower, upper quartile) vs. median day 31 (=post) (lower, upper quartile): 18(15, 21) vs. 19(16, 21), p = 0.24). I.e., the surgery alone and the recovery time of one week after each surgery had no impact on the AM discrimination performance of the animals. The same was true for group LR (Figure 4A, center panel): Friedman-ANOVA indicated no significant deficit in AM discrimination in the 6-day DP (ANOVA Χ^2^ (48, 2) = 3.30, p = 0.19) and Wilcoxon test for single day DP around each lesion (1^st^ lesion pre vs. post: 18(15, 22) vs. 18(14, 23), p = 0.72; 2^nd^ lesion pre vs post: 18(15, 22) vs. 16(12, 21), p = 0.16). We also did not find any significant changes of 6-day DP within the 15 days of training between the two lesions when comparing the first and last 6 day bins (Wilcoxon test: sessions 16 to 21 median: 18(14, 23) vs. sessions 25 to 30 median 18(14, 22), p = 0.78). This was also true for the 6-day DP after the 2^nd^ lesion and the 6 day DP at the end of training (Wilcoxon test: sessions 31 to 36 median: 16(12, 21) vs. sessions 40 to 45 median: 15(10, 21), p = 0.42). In other words, animals with both unilateral and bilateral lesion of the AC did not show any signs of impairment in DP when removing the left AC first and this effect was constant over the whole training period.

On the other hand, when investigating the 6-day DP of group RL animals (Figure 4A, right panel) Friedman-ANOVA indicated a significant difference between final DP (days 10 to 15) and the DP at the first 6 days after each lesion (Χ^2^ (42, 2) = 6.33, p = 0.04). Detailed analysis showed that this effect did not result from the first lesion (right AC lesion; Wilcoxon test: sessions 10 to 15 median: 21(17, 23) vs. sessions 16 to 21 median: 20(16, 23), p = 0.75) but from the second lesion affecting the left AC (Wilcoxon test: sessions 10 to 15 median: 21(17, 23) vs. sessions 31 to 36 median: 18(12,21), p = 0.02). This was somewhat different for the single day DP around each lesion (Wilcoxon tests: 1^st^ lesion pre vs. post: 22(21, 25) vs. 18(16, 20), p = 0.02; 2^nd^ lesion pre vs post: 17(16, 25) vs. 20(11, 21), p = 0.40) where the first lesion had a significant impact on the DP but not the second. Independent of these minor differences between single and 6-day DP, we found neither a change of 6-day DP between both lesions (Wilcoxon test: sessions 16 to 21 median: 20(16, 23) vs. sessions 25 to 30 median: 19.5(17, 23), p = 0.71) nor between the 6-day DP after the 2^nd^ lesion and the end of training (Wilcoxon test: sessions 31 to 36 median: 18(12, 21) vs. sessions 40 to 45 median: 19(15, 23), p = 0.19). In other words, the DP for AM stimuli was not affected when removing the right AC first but impaired (DP dropped from 21 to 18 jumps of maximal possible 30 jumps) when additionally lesioning the left AC and the animals did not show a significant recovery until the end of training.

A further between-group analysis revealed no significant differences between the groups at each investigated time interval as indicated by Kruskal-Wallis ANOVAs (final DP (sessions 10 to 15 median): H(2, 138) = 5.18, p = 0.08; 1^st^ lesion (sessions 16 to 21 median): H(2, 138) = 2.11, p = 0.35; 2^nd^ lesion (sessions 31 to 36 median): H(2, 138) = 3.39, p = 0.18). So all animals were equally able to perform the task of fast AM discrimination independent of their lesion status.

Additionally to the investigation of DP we repeated the analyses described above using the d’ values calculated from CR+ and CR-. In general we found the same results as in the analysis of DP (Figure 4B, statistics are given in the panels). In control animals and in the LR group animals no change in d’ could be found at the three time intervals while in the RL animals a tendency for a reduced d’ value especially after the 2^nd^ lesion emerged. When investigating the changes between the sessions 16 to 21 vs. 25 to 30 and 31 to 36 vs. 40 to 45 for each group by t-tests, we found no differences in any of the groups. This was true for the control group (mean ± standard deviation: 1.77 ± 0.47 vs. 1.77 ± 0.50, p = 0.98 and 1.80 ± 0.49 vs. 1.67 ± 0.57, p = 0.23) and for both lesion groups (LR: 1.77 ± 0.65 vs. 1.85 ± 0.66, p = 0.56 and 1.61 ± 0.69 vs. 1.54 ± 0.71, p = 0.64; RL: 1.94 ± 0.59 vs. 2.09 ± 0.56, p = 0.24 and 1.75 ± 0.57 vs. 1.92 ± 0.76, p = 0.31). So comparable to the results of the DP analyses, we found a tendency for a performance reduction in AM discrimination after the second lesion when lesioning the right AC first and then the left AC without any significant recovery until the end of training. This tendency became significant when pooling both lesion groups (1-factorial ANOVA: F(2, 240) = 4.15, p = 0.017) and Tukey post-hoc tests showed the d’ values after the 2^nd^ lesion (sessions 31 to 36) to be significantly lower than before the 1^st^ lesion (sessions 10 to 15) with p = 0.013. Nevertheless, we did not find any significant differences when comparing the pooled lesion group with the C animals at the three different time points in a 2-factorial ANOVA (factor group: F(1, 367) = 0.43 p = 0.51; factor session: F(2, 367) = 1.42, p = 0.24; interaction: F(2, 367) = 1.90, p = 0.15).

In summary, despite the small discrimination performance loss in the pooled lesion group or in the RL group alone, respectively, animals of all groups were able to discriminate AM stimuli after ablation of both ACs independent of the analysis method. This was an unexpected result as animals that receive simultaneous bilateral lesion of both ACs show a complete loss of DP in this particular AM discrimination paradigm, independent of pre-training experience (cf. [25]).

To further characterize this surprising discrimination ability we analyzed the CR+ (hit), CR- (false alarm) and ITC (inter trial crossings, measure of activity) rate of the animals. In Figure 4C the CR+ and CR- median values are given for three different 6-day intervals for the three groups. In the control animals (left panel) we did not find any significant differences between the 6-day median values taken before and after the 1^st^ lesion and after the 2^nd^ lesion (CR+: Friedman ANOVA Χ^2^ (48, 2) = 2.56, p = 0.28; CR-: Friedman ANOVA Χ^2^ (48, 2) = 2.75, p = 0.25) which was also true for the Bonferroni corrected Wilcoxon tests performed between all three time intervals (always p > 0.05). In both lesion groups we found significant differences in CR+ and CR- data with a parallel temporal development: Both CR+ and CR- increased over time as indicated by Friedman ANOVAs (Figure 4C, center panel group LR; CR+: Χ^2^ (48, 2) = 10.81, p = 0.004; CR-: Χ^2^ (48, 2) = 23.01, p < 0.001. Right panel group RL; CR+: Χ^2^ (42, 2) = 6.67, p = 0.036; CR-: Χ^2^ (42, 2) = 17.78, p < 0.001). Additional between group comparisons (C, LR and RL) by Kruskal-Wallis ANOVA for each time interval showed no significant differences in CR+ or CR- before or after the 1^st^ lesion, but became significant after the 2^nd^ lesion for both CR+ and CR-, indicating that both lesion groups made more CR+ jumps than control animals (H(2, 138) = 6.34, p = 0.04). Note that the multiple comparison of mean ranks post-hoc tests was not significant between single lesion groups and the control group, but pooled lesion group vs. control group were significantly different from each other in the Mann–Whitney U-test with p = 0.04. Nevertheless, in the between groups comparisons only LR lesioned animals showed a significantly higher false alarm rate (CR-) compared to C animals (H(2, 138) = 6.62, p = 0.037; multiple comparison of mean ranks LR vs. control: p = 0.034).

Figure 4D finally depicts the ITC rates of the animals of the three groups. Again the Friedman ANOVA indicated no significant changes in the control group (left panel: Χ^2^ (48, 2) = 5.09, p = 0.08) while Bonferroni corrected Wilcoxon tests (p = 0.048) indicated an increased activity after the 2^nd^ lesion when compared with pre lesion values. Both lesion groups on the other hand showed an increased ITC rate (center panel, group LR: Χ^2^ (48, 2) = 18.55, p < 0.001; right panel, group RL: Χ^2^ (42, 2) = 14.83, p < 0.001) which was especially enhanced after the 2^nd^ lesion as indicated by Bonferroni corrected Wilcoxon tests when compared with the values before and after the 1^st^ lesion (cf. Figure 4D). None of the Kruskal-Wallis ANOVAs at the three different time intervals showed significant differences between the three groups.

Taken together, these last analyses show that removing one AC, regardless of its side, already has effects on hit (CR+) rate, false alarm (CR-) rate but not on inter trial crossing (ITC) activity. Removing the second AC increases especially the CR- rates as well as the ITC rate. The increase of CR + counteracts the increase of CR- after the first lesion but cannot fully compensate the further increase of jumping affinity after the second lesion which leads to a slight drop in performance.

## Discussion and Conclusions

A number of studies have addressed the impact of bilateral auditory cortex lesions on auditory processing and perception. In humans, ablations of the auditory cortices in both temporal lobes are rare [28] and usually caused by multiple ischemic insults of the respective brain regions rather than head injury induced traumata (cf. [29]). In these cases patients often suffer from cortical deafness [28-38], although the patients’ specific impairment depends on the exact location and extent of the lesion [33,35] and possible retrograde fiber degeneration caused by the AC lesion [36]. In cases of complete cortical deafness patients are usually able to react to environmental sounds despite being unaware of hearing them [30]. This phenomenon can be explained by the fact that cortical deaf patients usually display normal auditory brainstem functions, e.g. normal BERA thresholds [29,37,38], despite profound to complete sensori-neural hearing loss on pure tone audiometry.

In animal models effects of bilateral AC lesion seem to be comparable to those observed in humans: It has been demonstrated in behavioral experiments that intact AC seems to be crucial for the discrimination of complex auditory sounds like complex tones with a certain pitch [39], species-specific vocalizations [40], or fast AM tones likes those used in this report [25]. In contrast, simple tasks like pure tone discrimination or discrimination of slow AM tones are possible without AC [25,41] and therefore may rely on normal or only slightly affected auditory brainstem processing [42]. In our animal model, this normal auditory brainstem processing is reflected in unchanged hearing thresholds (cf. Figure 1), as our control method (prepulse inhibition of the auditory startle response) depends on subcortical reflexes [43,44].

In the present report, the most intriguing and unexpected result is that there seems to be a fundamental difference in auditory impairment induced by sequential bilateral AC lesion compared to simultaneous bilateral lesion: We could demonstrate in an earlier study that simultaneous ablation of ACs in both hemispheres leads to a complete loss of the ability to discriminate fast AM tones even after pre-training prior to the lesion (Figure two in [25]). A sequential ablation of the two ACs with ongoing behavioral training between lesions as conducted in the present report has only very subtle, quantitative effects on discrimination performance (cf. Figure 4) and does not disable the ability of the animals to discriminate such sounds (cf. Figure 3). Note that the fact that AM discrimination in our paradigm is possible without AC does not imply that AC is not used for the task if it is intact. The slight drop in DP and d’ in both lesion groups can be explained by the increase in CR- that is stronger than the increase in CR+, especially after the second lesion. Both observations point to a generally higher activity state of the animals after the ablation of the second AC that is also reflected in increased ITC. These effects do not seem to be a result of the surgery itself, as sham control animals showed almost no changes in these parameters. It has been reported that cortical lesions can lead to neuronal disinhibition and hyperactivity (for review see [45]) which could explain the heightened bias to jump over the hurdle. Independent of the exact neuronal state of the animals, for the explanation of our main finding – the ability to discriminate fast AM without an AC – there must be some neuroplastic compensatory mechanisms triggered by or after the first, unilateral AC lesion. This compensation obviously enables the animal to continue to perform on a high level in the discrimination task even after the second AC is lesioned. Apparently, these neuroplastic changes occurring in the time between the two lesions do not take place in the contralateral AC, at least not exclusively, because in that case the ability to perform above chance in the discrimination task would get lost after the lesion of this contralateral AC. Interestingly the animals in this study do not show an increase (right AC lesion) or decrease (left AC lesion) in performance dependent on the side of the lesion as described in one of our recent studies [26]. This difference is most probably due to the pre-training of the animals in the present report which was not performed in the aforementioned other work. The auditory cortex seems to need training to be able to induce neuroplastic changes elsewhere in the brain to compensate for lost functions.

One may now speculate if these compensatory neuroplastic changes triggered by unilateral AC lesion affect subcortical auditory centers or higher cortical areas. A simple view would suggest that, after AC finally is bilaterally destroyed, projections to higher cortical areas containing auditory information should be disrupted. In this view, subcortical processing, where the auditory input from the periphery is still available, may be responsible for the retained discrimination performance. Nevertheless, numerous data demonstrate that sensory processing is not that simple and involves structures outside the sensory pathways, for example frontal and prefrontal areas, limbic structures, temporal association cortices, cerebellar structures (cf., e.g., [30,31]) or the habitual learning system (for review see [46]). The observation that patients with cortical deafness still show long-latency auditory event-related potentials [30,37] might point into this direction.

Although it is currently not clear whether the compensatory mechanism triggered by the first unilateral AC lesion suggested in this study is also present in humans [34], our result may still open new perspectives for the treatment of patients with AC damage: As most patients suffer from unilateral AC damage first – as our animal model – while cortical deafness due to the lesion of the second AC usually develops after a second ischemia [28,34], this opens a time window for potential prophylactic treatments to specifically trigger compensatory neuroplasticity. Whether such a treatment like the auditory discrimination training applied in our animal model is really needed to trigger such compensatory neuroplasticity will have to be investigated in future studies.

## Methods

### Animals

Thirty-one adult (12 to 39 weeks old), male Mongolian gerbils (*Meriones unguiculatus*) weighing 60–110 g were used in this study. All animals were purchased from Janvier (Janvier, Saint Berthevin Cedex, France) and kept at a room temperature of 22 to 24°C at 50 to 60% relative air humidity under a 12 h / 12 h light–dark cycle. All experiments were conducted in accordance with the NIH Guidelines for Animals in Research and with the ethical principles defined by the German law for the protection of experimental animals. The experimental protocols were approved by the state of Bavaria (Regierungspräsidium Mittelfranken, Ansbach, Germany; registration number 54–2532.1-32/09).

### Behavioral audiometry

Animals were controlled for normal hearing abilities (pure tone audiometry) prior to and after the final experiments using prepulse inhibition (PPI) of the acoustic startle response (ASR) as described in detail elsewhere [47]. In short, audiograms were measured using pure tones ranging from 0.5 to 16 kHz in one octave steps with a duration of 6 ms including cos^2^ rise and fall times of 2 ms in a pseudo-randomized order. The startle stimuli were set to 90 dB SPL, intensities of prepulse stimuli ranged from 0 to 50 dB SPL in 10 dB steps and preceded the startle stimulus by 100 ms. Prepulse stimuli had the same frequency and duration as the startle stimulus. The animals were placed in a transparent acrylic tube (length: 10 cm; inner diameter 4.3 cm) with a stainless steel grate (wire mesh width 0.5 mm) on the front and a door on the rear side. This tube was positioned 10 cm from a speaker (Canton Plus X Series 2) onto a Honeywell FSG15N1A piezo sensor, assembled within an IAC acoustic chamber (IAC, Niederkrüchten, Germany) on a TMC low-vibration table. Stimulus generation and data acquisition was controlled using custom-made Matlab 2008 programs (MathWorks, Natick, MA, USA; stimulation/recording sampling rate 20 kHz). For sound generation the frequency response function of the speaker was calibrated to produce an output spectrum that was flat within +/− 1 dB. All stimuli were repeated 15 times and the data were checked offline by eye; trials in which the animals moved within a time window of 100 ms ahead of the startle stimulus were rejected and not used for further analysis. The response data over all prepulse intensities of each frequency were fitted by a sigmoidal Boltzmann function and the inflection point of this function was set as hearing threshold of the frequency [48]. Pre and post lesion/training thresholds were compared using 2-factorial ANOVA (cf. Figure 1).

### Behavioral discrimination training

Animals were trained in a two compartment shuttle-box (Coulbourn Instruments, Whitehall, USA) placed in an acoustic chamber (IAC, Niederkrüchten, Germany) using a foot-shock-motivated avoidance GO/NOGO procedure as described earlier [49] Electrical foot shocks applied through the floor grid served as unconditioned stimulus (UCS). Gerbils were trained to discriminate between fast 100% sinusoidal amplitude modulated tones (AM) with modulation frequencies of either 160 or 320 Hz, but identical carrier frequencies of 2 kHz (i.e., two completely modulated 2 kHz pure tones modulated by either 160 or 320 Hz). The auditory stimulation was provided via two speakers in the ceiling of the shuttle-box about 20 cm above the floor grid. The stimuli had 400 ms duration with 5 ms rise and fall times and were presented repeatedly at 2 Hz with 60 dB SPL, as measured with a Brüel & Kjaer Precision Sound Level Meter 2235 connected to a Brüel & Kjaer Standard Microphone 4176 (Brüel & Kjaer, Bremen, Germany) in 15 cm distance from the speaker (note that due to reflections within the shuttle-box the sound intensity may vary with location).

Gerbils were trained in daily sessions to discriminate periodicities of 160 Hz vs. 320 Hz, a training paradigm that has been shown to be dependent on an intact auditory cortex (cf. [25]). The lower periodicity served as the conditioned stimulus (CS+; conditioned stimulus followed by the foot shock UCS) that required jumping (GO) across the hurdle (height: 4 cm), whereas the higher periodicity served as the conditioned stimulus (CS-; conditioned stimulus not followed by the UCS) that required abstaining from jumping across the hurdle (NOGO). Within each training session, 30 trials of each stimulus (CS+ or CS-) were presented in pseudo-randomized order. Variable intertrial intervals (start to start) were 16 +/− 4 s. Crossings of the hurdle during a 4 s presentation of the CS+, i.e. correct conditioned responses (CR+) and false alarms during the 4 s presentation of the CS- (CR-), respectively, were counted in each session. If the animal did not cross the hurdle within 4 s after the onset of the CS+, the UCS was turned on and the CS+ presentation continued until the gerbil crossed the hurdle, but maximally for another 4 s. An additional UCS (duration 0.5 s) was applied on the other side of the hurdle upon false alarm to the CS-. Strength of the UCS ranged between 150 and 450 μA and was adapted individually to each animal to be unpleasant (search for escape) but not painful to avoid fear behaviour (freezing). Polarity of the steel bars of the floor grid was varied randomly for each trial to avoid learning of the polarity pattern of the floor grid by the animals.

**Figure 5:**
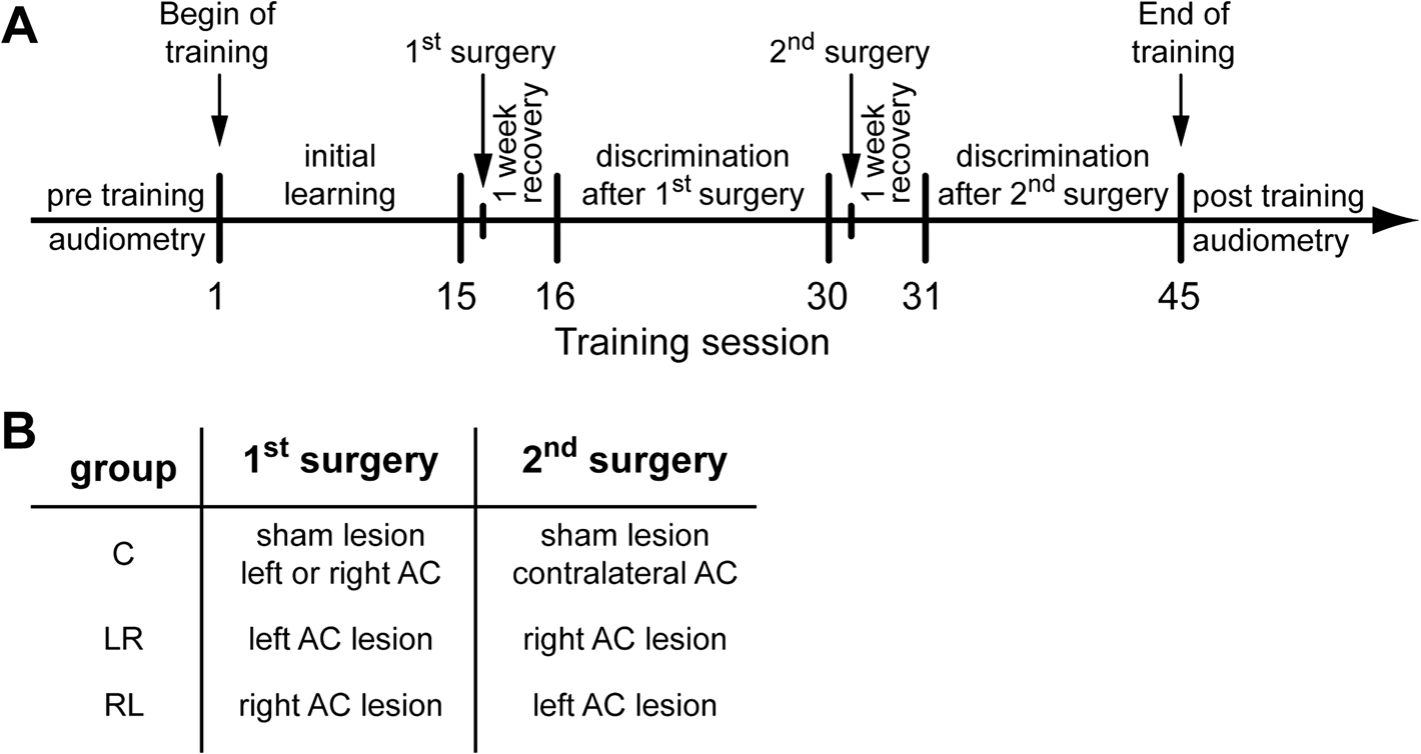
**Overview of experimental design. A** Experimental scheme: Temporal sequence of the experiments. **B** Table of group treatment.

Prior to cortical ablation (cf. below) all animals received 15 days of training with one training session per day. After this initial training phase, animals were divided in sub-groups that received auditory cortex (AC) ablation on the right or left side or sham surgeries, respectively. Sham-lesioned animals with complete surgery but without cortical ablation served as controls. Because there were no significant differences in the learning performance of left or right sham-lesioned animals (Mann–Whitney U-tests on a daily basis, always p > 0.05), these were combined into one single control group, consisting of equal amounts of left (n = 4) or right (n = 4) sham-lesioned animals. After surgery and one week recovery the animals received another 15 days of training with again one training session per day. Following this second training phase all animals received a second surgery with or without ablation of the second AC: Left-lesioned animals received ablation of right AC, right-lesioned animals received ablation of left AC, and sham-lesioned animals received another sham surgery contralateral to the side of the first. Following the second lesion and one week recovery the animals were trained for the final 15 days. For an overview of this experimental scheme refer to Figure 5A and B.

This procedure resulted in a total of 3 experimental groups in which only animals with successful surgeries were included (cf. below): left-right sequential AC lesion (group LR, n = 7), right-left sequential AC lesion (group RL, n = 7) and sham-lesioned control group (group C, n = 8), all trained to discriminate 160 vs. 320 Hz periodicity.

### Cortical ablation

Surgery was performed as described earlier [20,21,25,41,50] under deep general anesthesia by subcutaneous infusion of ketamine (96 mg/kg Ketamin, Ratiopharm, Ulm, Germany), xylacine (4 mg/kg Rompun 2%, Bayer, Leverkusen, Germany), atropine (1 mg/kg Atropinsulfat, B.Braun Melsungen AG, Melsungen, Germany) and isotonic sodium chloride solution (Berlin-Chemie AG, Berlin, Germany) in a mixture of 9:1:2:8 at a rate of 0.1 to 0.15 ml/h, after an initial dose of 0.2 to 0.3 ml. Body temperature was maintained at 37°C using a remote-controlled heating blanket. For cortical ablation the skin over the temporal bone on the ablation side was cut and retracted, the musculature covering the temporal bones was partly removed. The AC was then exposed by craniotomy and thermo-coagulated using a fine tip soldering iron (Ersa, Wertheim, Germany) centered to the primary AC (field AI). Using well described landmarks of the cortical vasculature [27,51] the lesion attempted to cover AI and the surrounding fields of gerbil AC which comprise an area of approximately 8 to 9 mm^2^. Finally the remaining musculature was repositioned and the skin was sealed using suture material (Ethilon, Polyamid6, Johnson & Johnson, Neuss, Germany) and Histoacryl (B.Braun, Melsungen AG, Melsungen, Germany). For post-operative pain reduction one dose of Metamizol (15 mg Novaminsulfon, Ratiopharm, Ulm, Germany) was s.c. administered. Ablation surgery of the sham-lesioned group was conducted identically as described for the lesioned groups but without thermo-coagulation. Animals were given a recovery time of two days before behavioral training started. By that time the animals showed no sign of any impairment and normal behavior.

### Estimation of lesion volume

To determine volume and position of cortical lesions gerbils received structural *In Vivo* MRI scans after each cortical ablation (cf. Figure 2). This method has been demonstrated to yield results similar to post-mortem histological analysis (cf. [50]) and was chosen here to be able to evaluate proper lesion size and location after the first, unilateral lesion already, prior to additional training. For this, animals were anaesthetized with 1.5–2% isoflurane (in 70:30 N_2_O:O_2_; volume ratio) and secured using a head-holder with bite bar to reduce motion artifacts. MRI measurements were performed on a 4.7 T BRUKER Biospec scanner, horizontal magnet with 40 cm free bore and gradient strength of 200 mT/m, equipped with an actively RF-decoupled coil system and a 3 cm quadrature surface receiver coil, located directly above the head of the animal to maximize signal-to-noise. In order to minimize scan time, only 22 coronal slices covering the dorso-ventral extension of the lesion were scanned with 2D T2 weighted spin echo sequence (slice thickness 0.3 mm, field of view 25 • 25 mm, matrix 256 • 256, in plane resolution 0.098 mm/pixel, TR = 2649.1 ms, TE_eff_ = 56 ms, bandwidth 50 kHz) using a RARE sequence (RARE factor 8) [52] resulting in a total measurement time of 28 min, 15 sec.

The contour of the lesion was determined manually (cf. Figure 2E and F), the lesioned outer cortical border was interpolated from the remaining cortical surface. Lesion volume was computed by Amira (FEI Visualization Sciences Group, Düsseldorf, Germany). Only animals where the lesion was restricted to the cortex with no lesion of adjacent structures like the hippocampus were included in the study.

### Statistical analysis of behavioural data

Kolmogorov-Smirnov tests rejected normal distributions of the learning parameters in all animal groups with p < 0.05, therefor we analysed all data statistically with nonparametric tools. To quantify the behavioural learning data we used measures of how fast and how well gerbils learned to discriminate between CS+ and CS-: As an index for learning speed (LS) we determined for each individual animal, the first session in which the responses to the CS+ (hits) were significantly different from those to the CS- (false alarms) on the 1% level (fourfold table χ2-test). Responses were hit (hurdle crossing in response to CS+), miss (no response to CS+), false alarm (hurdle crossing in response to CS-) and correct rejection (no response to CS-). This session had to be followed by a session that also fulfilled this criterion.

The discrimination performance (DP) was calculated on a daily basis subtracting CS- from CS+. We determined a measure of the final discrimination performance (final DP) after the initial training (i.e., before first surgery) which is the median of the difference between CR+ and CR- that was reached during the last 6 training sessions by each individual gerbil. We chose the last 6 sessions because Friedman-ANOVAs showed no further changes in DP in all groups during these sessions. We compared the training data of the first 6 days after both lesions (6-day DP) with this final DP to identify any discrimination performance differences relative to the healthy animals. Generally, within one group we performed either Wilcoxon tests or Friedman ANOVAs. Between two independent groups all parameters were tested by two-tailed Mann–Whitney U-tests and between several independent groups with Kruskal-Wallis ANOVA. We repeated the analyses with parametric tests on the sensitivity index d’ values calculated from CR+ and CR- hurdle jumps, where d’ = Z(CR+) - Z(CR-) [53-56]. Additionally we investigated CR+, CR- and the inter trial crossings (ITC, measure of activity) using the mentioned nonparametric tests to asses not only the DP and d’ values but also the absolute values of hit and false alarm rates.

## Abbreviations

AC: auditory cortex; AM: amplitude modulation; ASR: auditory startle response; C: control group; CR+ / CS+: conditioned response/conditioned stimulus followed by UCS; CR- / CS-: conditioned response/conditioned stimulus not followed by UCS; d': discrimination sensitivity index; DP: discrimination performance; LR: lesion group: first left, second right lesion; LS: learning speed; MRT: magnet resonance tomography; PPI: prepulse inhibition; RL: lesion group: first right, second left lesion; UCS: unconditioned stimulus.

## Competing interests

The authors declare that they have no competing interests.

## Authors’ contributions

MD performed all experiments and assisted in data analysis. AH provided access to the MRT and assisted in lesion data analysis. KT supervised the behavioral data analysis and wrote a part of the manuscript. HS designed and coordinated the study and helped to draft the manuscript. All authors read and approved the final manuscript.
